# Design and use of an ex vivo peripheral simulating bioreactor system for pharmacokinetic analysis of a drug coated stent

**DOI:** 10.1002/btm2.10618

**Published:** 2023-11-02

**Authors:** Danyi Chen, Colin Krinsky, Mollie Phillips, Catherine Allred, Ava Khan, Linda B. Liu, Uwe Christians, Saami K. Yazdani

**Affiliations:** ^1^ Wake Forest University Department of Engineering Winston‐Salem North Carolina USA; ^2^ iC42 Clinical Research and Development University of Colorado Aurora Colorado USA

**Keywords:** bioreactor, drug delivery, ex vivo, paclitaxel, peripheral arterial disease, pharmacokinetics, stent

## Abstract

Currently, there are no ex vivo systems that can model the motion of peripheral arteries and allow for the evaluation of pharmacokinetics (PK) of endovascular devices. The objective of this study was to develop a novel peripheral simulating bioreactor system to evaluate drug pharmacokinetics of stents. We utilized 3D‐printed and off‐the‐shelf components to construct a peripheral‐simulating bioreactor system capable of mimicking the motion of peripheral arteries. Servo motors were primarily used to shorten/elongate, twist, and bend explanted porcine carotid arteries. To evaluate the pharmacokinetics in the bioreactor, drug‐eluting stents were deployed within explanted arteries and subjected to vascular motion along with pulsatile flow conditions. Following 30 min and 24 h, the arteries were removed, and paclitaxel levels were measured. Scanning electron microscopy was also performed to evaluate the stent surface. Arterial paclitaxel levels of the stent‐treated arteries were found to be higher at 30 min than at 24 h following pulsatile and no vascular motion and even higher at 24 h following pulsatile flow and vascular motion. The residual drug on the stent significantly decreased from 30 min to 24 h. Scanning electron microscopy confirmed the loss of paclitaxel coating at 24 h and greater disturbance in stents under peripheral motion versus pulsatile only. This system represents the first ex vivo system to determine the PK of drug‐eluting stents under physiological flow and vascular motion conditions. This work provides a novel system for a quick and inexpensive preclinical tool to study acute drug tissue concentration kinetics of drug‐releasing interventional vascular devices designed for peripheral applications.

**Translational Impact Statement**

Drug eluting stents (DES) have become the gold standard to treat cardiovascular disease. As we continue develop safer and more effective DES, in particular for peripheral applications, our ability to assess and evaluate these devices are critical. This work provides a novel bench‐top system that can model the motion of peripheral arteries and allow for the evaluation of stent pharmacokinetics. This system provides both a valuable alternative method to traditional animal testing and aid in identifying key design and delivery parameters of medical devices.

## 1 INTRODUCTION

Peripheral Artery Disease (PAD) is an atherosclerotic disease affecting approximately 12% of the adult population and over 20% of adults older than 70.^1^ PAD is caused by a buildup of cholesterol plaques on the inner walls of peripheral arteries, reducing blood flow to the arms and legs.^2^, ^3^ Patients afflicted can experience intermittent claudication (IC), repeated hospitalizations, and limb loss that contributes to lower quality of life and high rates of depression.^4^ Even asymptomatic patients suffer poorer quality of life and functional performance compared with the same age groups.^5^ Patients with PAD are also more susceptible to myocardial infarction, stroke, cardiovascular death, and higher all‐cause mortality rates.^6^, ^7^, ^8^

Endovascular intervention with either stenting or balloon angioplasty, is currently the preferred treatment option for PAD. However, restenosis and stent fracture remain a problem, leading to poor and repeated procedures for patients.^9^, ^10^ The treatment challenge of PAD, as compared to coronary artery diseases, is that peripheral arteries are subjected to repeated external mechanical stresses including flexion, shortening, and torsion.^11^ These vascular‐specific motions have been indicated to cause stent fracture, leading to higher rates of thrombosis and restenosis.^12^, ^13^, ^14^, ^15^

Stents designed to treat PAD are typically made from flexible nitinol material, allowing for more flexibility inside the arteries to withstand peripheral motion. Therefore, nitinol stents are less prone to fracturing than balloon‐expandable stainless steel or cobalt chromium stents designed for coronary arteries. Additionally, newer peripheral stents are coated with the anti‐proliferative drug paclitaxel, designed to elute the drug from the stent surface into the arterial wall. In recent years, clinical studies have shown that self‐expanding, drug‐eluting stents (DES) have lower restenosis and stent fracture rates than traditional bare metal stenting or balloon angioplasty.^16^, ^17^

The fundamental approach of DES relies on their ability to deliver a drug, typically paclitaxel, directly to the lesion site.^18^, ^19^ Pharmacokinetic evaluation of the target tissue is therefore performed to quantify tissue drug concentration to verify DES performance. Typically, these studies are performed in vivo since there is a lack of clinically relevant ex vivo models capable of testing the pharmacokinetic performance of DES in a biologically relevant model of the periphery. This study aimed to design a novel peripheral‐simulating ex vivo bioreactor system capable of evaluating the drug pharmacokinetics of DES.

## 2 METHODS AND MATERIALS

### 2.1 Vessel harvest and bioreactor system

Purchased harvested porcine carotid arteries from local abattoirs were rinsed in sterile PBS, and the excess fat, connective tissue, and fascia were dissected and removed. Vessels with a wall thickness of 1.0–1.2 mm and an inner diameter ranging from 3.8 to 4.4 mm were cut into 7–8‐cm segments and stored in 15‐mL centrifuge tubes at −20°C until needed. Frozen vessels were thawed and placed into the bioreactor system to be studied.

The peripheral simulating bioreactor was composed of a flow system and an actuating system. The flow system consists of a gear pump (Ismatec, Cole‐Parmer, Vernon Hills, IL) a flow reservoir, a distal flow restrictor, and two flexible vessel housing compartments made from silicone. The bioreactor flow medium consisted of DMEM containing low glucose [1000 mg/L], 4.0 mmol/L L‐glutamine, 110 mg/L sodium pyruvate, pyridoxine hydrochloride, 10% fetal bovine serum (Gibco), and 1% antibiotic‐antimycotic (Gibco). The total volume of medium used in the system was 350 mL. The bilateral design allowed the evaluation of two vessels simultaneously. The flow rate and pressure were monitored via an ultrasonic flow meter (Transonic Systems Inc., Ithaca, NY) and a catheter pressure transducer (Millar Instruments, Houston, TX).

**FIGURE 1:**
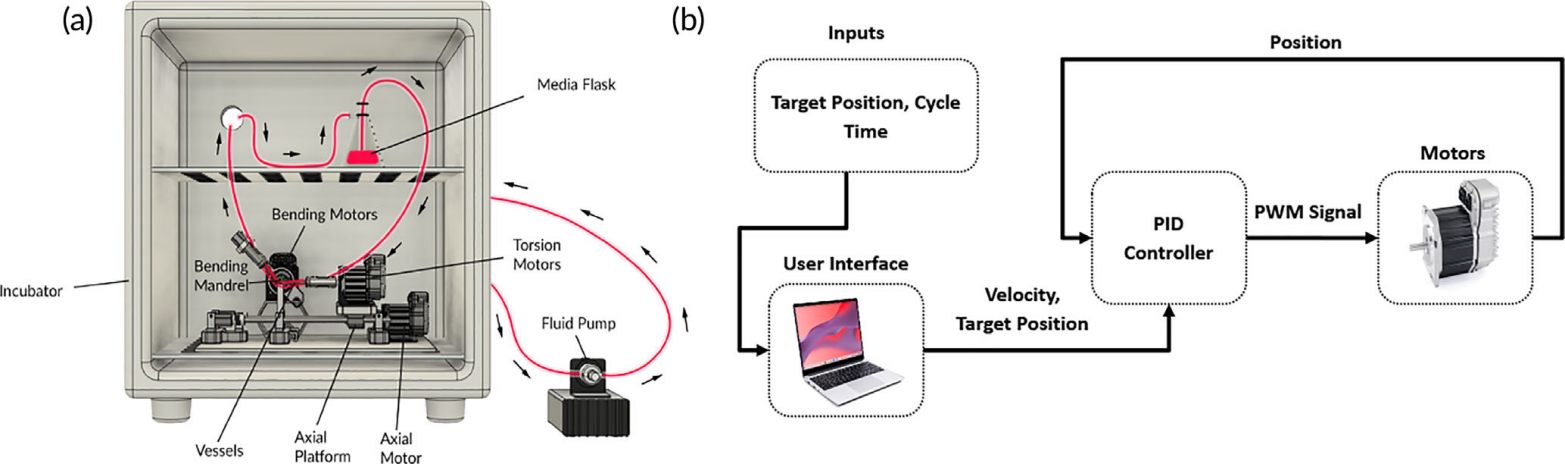
Schematic representation of experimental setup for bioreactor system. (a) Overview of the system showing the incubator, peripheral simulating bioreactor, and flow pump. (b) The user specifies the amplitude, cycle time, and experiment length for each motion (twist, bend, shorten) through the high‐level controller. This information is then transmitted to the low‐level controller, which incorporates position feedback using an integrated PID controller.

The 3‐degree‐of‐freedom actuating system was constructed from custom 3D‐printed and off‐the‐shelf components. Movement of the vessels is controlled via five 75 W Teknic Clearpath‐SCSK integrated DC servo motors with peak torque outputs of 0.5 Nm (Figure 1). Two motors are axially mounted to each vessel to apply torsion force; two motors are mounted perpendicular to the vessels to bend the vessels around a removable mandrel, and one motor actuates a linear drive to apply axial shortening simultaneously to all vessels. The range of motion for the twist, bend, and shorten‐elongate motions is as follows: 0°–180°, 0°–90°/5–300 mm radius, and 0–100 mm, respectively. The peripheral simulating bioreactor operates in cycles. In each cycle, the motors apply the flexion, torsion, and compression forces over a fixed time interval; then, the motors reverse back to the starting position over the same interval. The system then holds the position until the next cycle activates. The cycles aim to simulate any natural human movements during the day, for example, a walking stride, sitting down, or climbing stairs.

The control system used for the peripheral simulating bioreactor is a joint space closed‐loop speed controller. The input variables are the positional amplitude in all three degrees of freedom, the cycle time, and the total operating time. The system architecture is presented in Figure 1. The system uses positional feedback from built‐in encoders in the motors to validate that the system has completed a movement cycle.

### 2.2 Stent delivery and experimental conditioning

**FIGURE 2:**
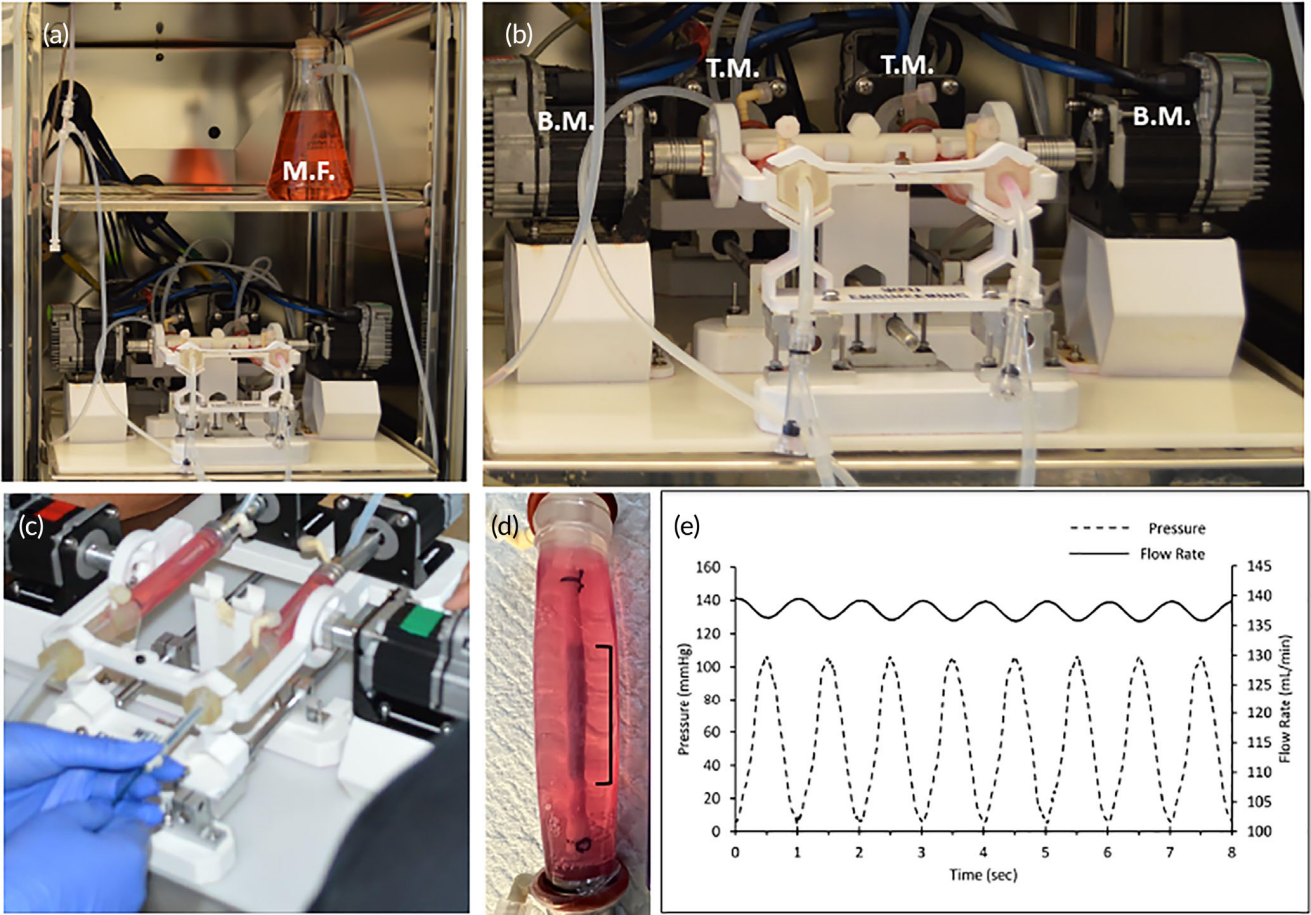
Peripheral simulating bioreactor. (a) Operational view of the peripheral simulating bioreactor within the CO_2_ incubator. (b) Close‐up view of the peripheral simulating bioreactor showing the parallel configuration. (c) Deployment of a stent within an artery. The stent is deployed into the artery using endovascular techniques (guidewire). (d) Post‐deployment view of the stent within the artery. (e) Pressure and flow rate measurement of the bioreactor flow system. BM, bending motor; MF, medial flask; TM, torsion motor.

To test the feasibility of the peripheral simulating bioreactor system, Zilver paclitaxel‐eluting peripheral stents (Cook Medical, Bloomington, IN) were deployed within the harvested porcine carotid arteries and subjected to peripheral motion. Prior to delivery, a guidewire was positioned within the lumen of the artery. Following endothelial injury by balloon angiopathy, the diameter of the explant artery was measured by ultrasound. The Zilver stent was then deployed into the artery based on the manufacturer's delivery parameter recommendation (Figure 2). After 2 h of pulsatile‐only flow conditions, the arteries were subjected to peripheral motion (torsion: 2.1°/cm, axial shortening: 4.0%, and bending radius: 72.1 mm) to mimic the proximal/mid superficial femoral artery motion.^20^, ^21^, ^22^, ^23^ Ex vivo treated arteries (*n* = 3 per time point, evenly matched) and the Zilver stents were either harvested at 30 min (only pulsatile flow) or 24 h (only pulsatile flow or pulsatile flow and peripheral motion) post‐deployment for pharmacokinetic and scanning microscopy analysis. The choice to omit peripheral motion condition for the first 30 min was made on the basis of clinical relevance, as patients are typically not able to move their leg during the initial 30 min following stent deployment. During the trials involving peripheral motion, a total of 2880 motion cycles were applied, which were in proximity to the average daily steps taken by claudicating patients.^24^

### 2.3 Quantification of paclitaxel tissue concentrations and scanning electron microscopy

Paclitaxel stent and tissue concentrations were quantified using a validated, previously described high‐performance liquid chromatography (HPLC)‐electrospray ionization–tandem mass spectrometry system assay (LC–MS/MS).^25^, ^26^, ^27^

To visualize the stent surface following deployment, the 30‐min and 24‐h treated stents were fixed with 10% formalin and dehydrated in a graded series of ethanol. After critical point drying, the tissue samples were mounted and sputter‐coated with gold. The specimens were visualized using a Phenom XL scanning electron microscope (Thermo Fisher Scientific, Waltham, MA).

### 2.4 Statistical analysis

All values were expressed as mean ± standard deviation (SD). Continuous variables were compared between groups using one‐way analysis of variance (ANOVA) using GraphPad Prism 9 (GraphPad Software, La Jolla, CA, USA). A value of *p* ≤ 0.05 was considered statistically significant. If statistical significance was shown, a comparison of quantitative data of multiple groups was performed by Tukey's multiple comparisons post hoc test.

## 3 RESULTS

Figure 2 shows representative images of the DES being deployed within the porcine carotid arteries of the ex vivo bioreactor system. The stents were exposed to a physiological flow consisting of a mean flow rate of 134 mL/min and a mean pressure of 55 mmHg [see Figure 2e]. In the peripheral‐simulating 24‐h group (torsion: 2.1°/cm, axial shortening: 4.0%, and bending radius: 72.1 mm), a total of 2880 cycles were completed. Figure 2 shows the bioreactor within the cell culture incubator during the 24‐h cycle.

To evaluate tissue drug retention, treated arterial segments were removed 30 min and 24 h post‐stenting. Due to the optical clarity of the vessel housing, the treatment area could be clearly identified to ensure that only the portion of the artery treated by the drug was excised (Figure 2d).

**FIGURE 3:**
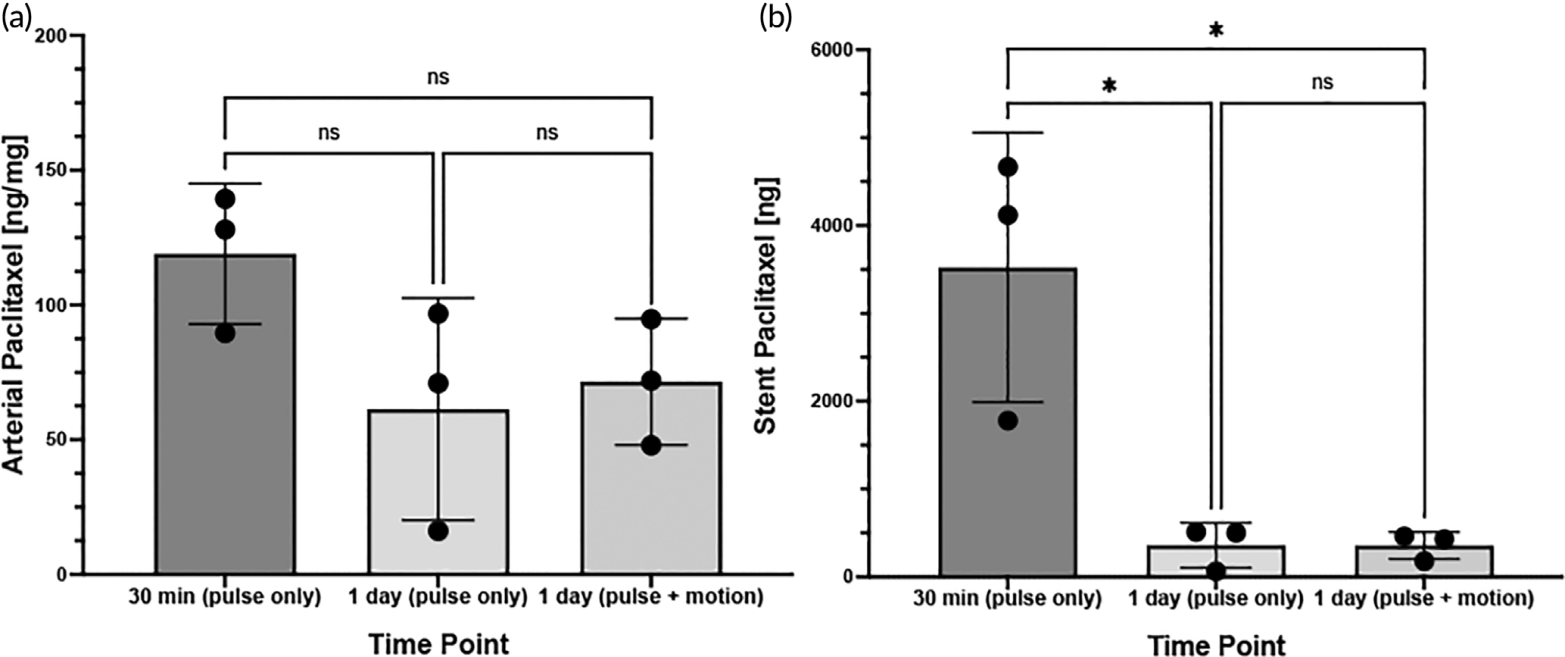
Paclitaxel analysis in treated artery tissue and remaining on stents. (a) Scatter plots displaying measured arterial paclitaxel concentrations within the drug eluting stent‐treated segments expressed as ng/mg from the 30‐min and 24‐h experiments. (b) Scatter plots displaying measured residual stent paclitaxel amount remaining on the deployed drug eluting stent‐treated specimens expressed as ng from the 30‐min and 24‐h experiments. Each bar represents the mean ± standard deviation. Data were analyzed using one‐way ANOVA. *p < 0.05, NS, not significant.

**FIGURE 4:**
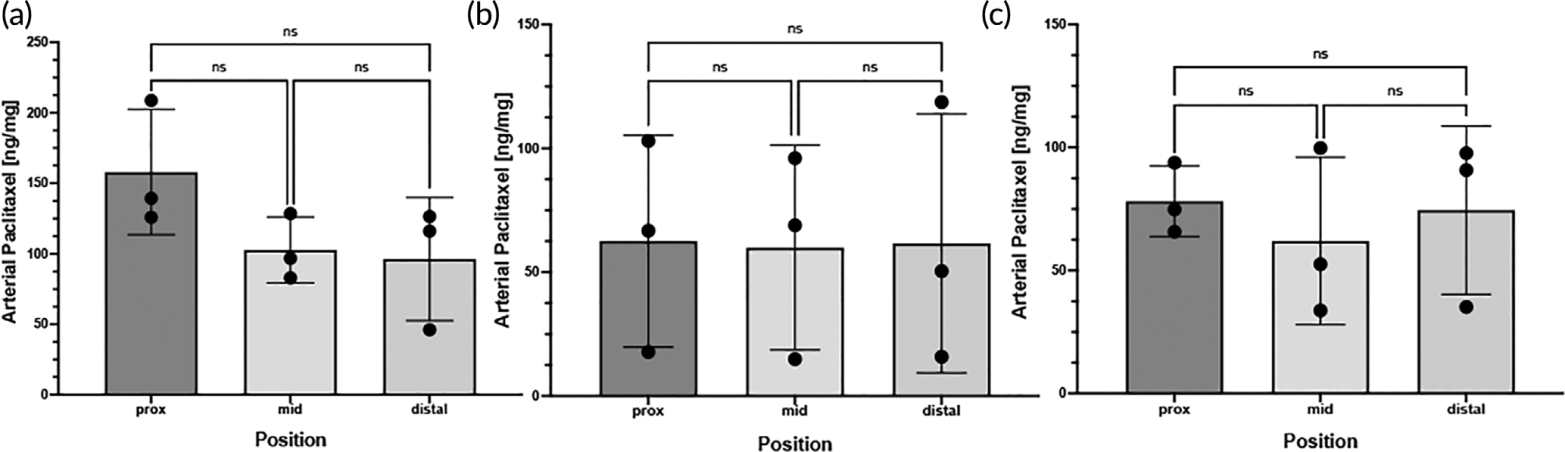
Pharmacokinetic analysis of the stented tissue. Scatter plots displaying measured arterial paclitaxel concentrations within the proximal, middle and distal segments of the drug eluting stent‐treated segments expressed as ng/mg from the (a) 30‐min, (b) 24‐h of pulsatile only and (c) 24‐h of pulsatile with vascular motion experiments. Each bar represents the mean ± standard deviation. Data were analyzed using one‐way ANOVA. *p < 0.05, NS, not significant.

Arterial tissue paclitaxel concentrations of the stented ex vivo pig arteries were measured at 119 ± 26.1 ng/mg at 30 min, 61.37 ± 41.2 ng/mg at 24 h under pulsatile flow only and 71.56 ± 23.4 ng/mg at 24 h under pulsatile flow and peripheral motion. Differences were not significant between the various time points (*p* = 0.13) (Figure 3a). We further quantified drug retention within the treatment lesions at the proximal, middle, and distal regions of the arteries and showed uniform distribution of the drug deposited by the stent (Figure 4).

**FIGURE 5:**
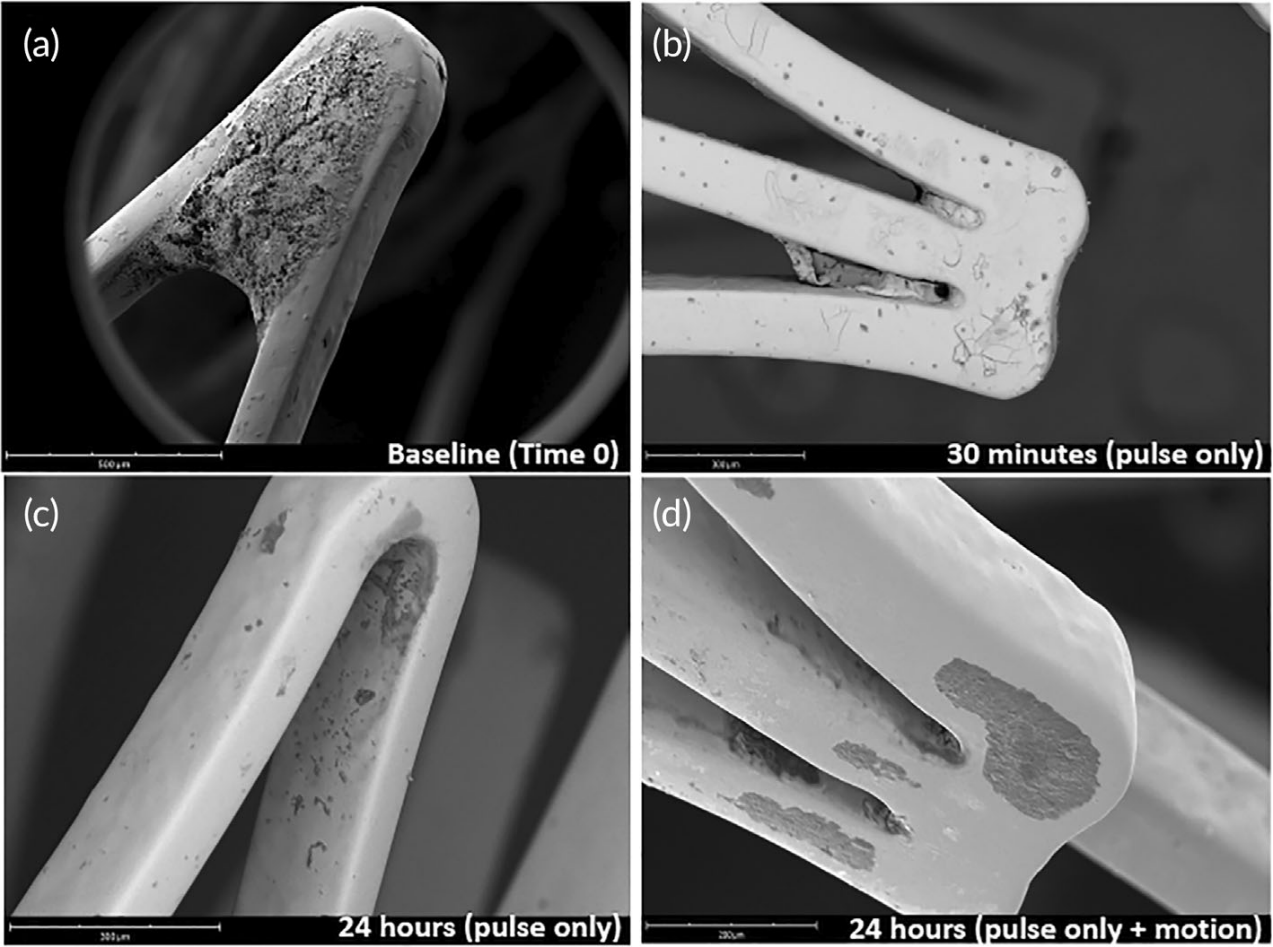
Scanning electron microscope images of implanted drug eluting stents. Representative SEM images the Zilver drug eluting stent (a) prior to deployment (baseline), (b) following 30‐min of pulsatile flow conditions, (c) following 24‐h of pulsatile flow conditions and (d) following 24‐h of pulsatile flow with vascular motion.

In addition to the tissue, we successfully quantified drug residual on the stent at the 30‐min and 24‐h time points. Quantification of the Paclitaxel amounts remaining on the deployed devices demonstrated significantly greater drug residual on the 30‐min implanted stents as compared to 24 h (30 min: 3522 ± 1534 ng vs. 24 h—pulsatile only: 359 ± 257 ng vs. 24 h—pulse + motion: 357 ± 154 ng, *p* = 0.0075) (Figure 3b). Scanning electron microscopy of the deployed stent shows drug reminisce at baseline (pre‐deployment) and at 30 min, whereas by 24 h, drug on the stent surface is no longer visible (Figure 5).

## 4 DISCUSSION

The study's main objective was to develop a peripheral simulating bioreactor system to evaluate drug pharmacokinetics of implanted drug‐coated stents. The system's design permitted the unique motion of peripheral arteries, including bending, twisting, and shortening‐elongating of an explanted carotid artery to be realized. The results demonstrate that off‐the‐shelf stents can be delivered successfully to native porcine arteries using intravascular techniques and evaluated under peripheral motion. In addition, pharmacokinetic analysis of the stented arteries demonstrated a reduction of arterial tissue drug levels from 30 min to 24 h, similar to trends reported in literature.^28^ These results demonstrate a novel platform for evaluating drug release kinetics of interventional vascular devices intended for the periphery in an ex vivo setting.

Peripheral artery disease (PAD) continues to be costly (~ $21 billion annually) and impacts millions of people yearly.^29^ Caused by atherosclerosis, plaque build‐up narrows and restricts blood flow causing pain, mobility loss, and poor wound healing. In PAD, stenting and balloon angioplasty interventions have focused on expanding the narrowed artery and restoring blood flow. More specifically, stents coated with antiproliferative drugs, such as paclitaxel and sirolimus‐based drugs, have been implemented to target and inhibit neointimal hyperplasia and restenosis.^30^, ^31^, ^32^ Success is achieved in 90%–95% of patients with coronary artery disease 6 months following intervention.^33^, ^34^, ^35^, ^36^ However, this has not translated well to PAD. This is partly due to the degree of disease in the periphery that can be diffused within the vasculature and the additional biomechanical stresses associated with peripheral arteries, which undergo severe motion such as twisting and bending.^37^, ^38^, ^39^, ^40^

Several cadaver and clinical studies have analyzed the biomechanical movement of arteries to quantify the complex deformation observed in the periphery, particularly the superficial femoral arteries and popliteal arteries.^20^, ^21^, ^41^ In general, vascular motion has been categorized by axial shortening and elongation, bending, and twisting. Typically, these changes have been defined by the normalized change in the arterial segment from the supine position to a certain degree of flexion associated with walking, sitting, and stair climbing. For walking, the simulated hip and knee bends are positioned at 70° and 20°, respectively. For stair climbing and sitting, hip and knee bends are at 90° and 90°, respectively. In terms of axial shortening, values have been reported to range between 0.4% and 29.5% for sitting and stair climbing and 0% and 21.5% for walking. Axial twisting has ranged from 0.1° to 6.3° twist per centimeter. Therefore, an 8 cm length artery would twist anywhere from 0.8° to 50.4°. The bending radius, defined as 1/curvature, was reported to vary between 13 and 206 mm. Altogether, these reported studies highlight the complex and wide range of motion within the periphery.^20^, ^21^, ^41^ The benchtop‐compatible peripheral simulating bioreactor is capable of implementing all the aforementioned motions.

The motivation for the current work was two‐fold. Firstly, we wanted to develop a model that could mimic the wide range of peripheral motion. The model would aid in a better understanding of arteries undergoing peripheral motion and their interaction with implanted devices such as stents. Secondly, we wanted to develop this technology to serve as an intermediate step to rapidly assess the pharmacokinetics of drug delivery devices while limiting animal usage and cost of testing. For any short‐term in vivo experiment (<7 days), months of preparation are needed to obtain approval from IACUC, order animals, quarantine, and schedule the procedure to implant and retrieve the device or tissue. Each step of this process (animal housing, staff support, operating table time) increases the cost and duration of the study. Using fresh swine arteries from local slaughterhouses minimizes costs and animal usage to nearly zero. Additionally, fresh swine arteries are readily available in abundant quantities, at relatively low cost.

Many commercially available products are designed to evaluate stents intended for the periphery. Dynatek Lab (Galena, MO) can examine radial fatigue to fracture of stents using deployed stents within plastic/silicone tubing by alteration in load, frequency, temperature, or pH. The ElectroForce 9400 Multiaxial Peripheral Stent (MAPS, Test Instrument, New Castle, DE) examines deployed stents in plastic/silicone tubing under dynamic bending, rotation, extension/compression, and radial distention. In general, most testing of the current stent testing, prior to animal work, is focused on static bend testing (three‐point bend test) and radial compression tests. However, these tests do not simulate all the mechanical stresses experienced by the peripheral arteries. The ex vivo bioreactor system described here provides the most realistic testing model to date, implementing both pulsatile flow and peripheral vascular motion in the design.

**FIGURE 6:**
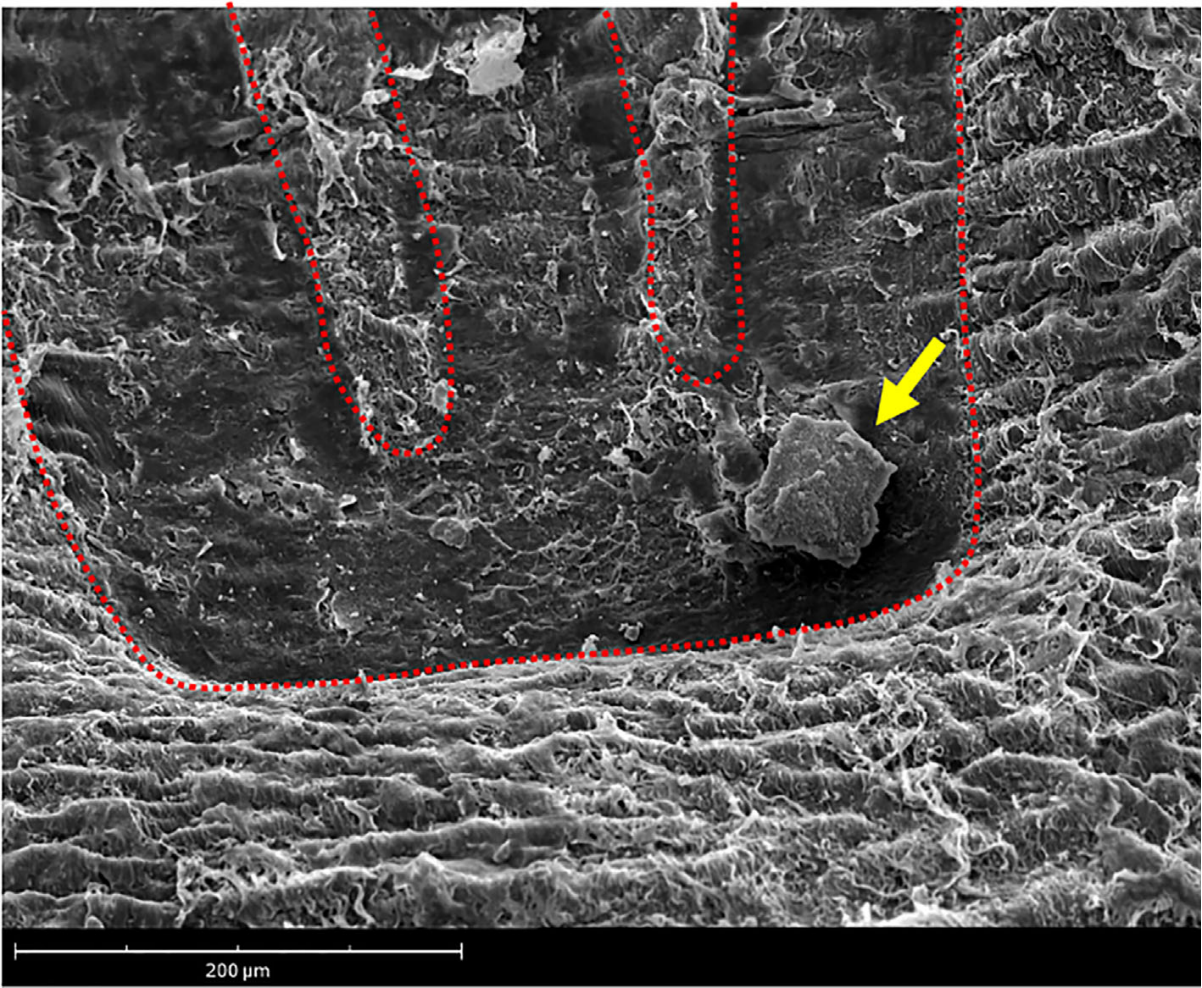
Electron microscope image of stented artery. A representative SEM image of a Zilver drug eluting stent treated artery. The red dashes indicate the presence of the stent strut within the intimal layer of the artery. The yellow arrow highlights paclitaxel drug adhered to the luminal surface, within the stent strut imprint.

Additionally, the use of explanted living native pig arteries as the test section allows quantifying and evaluating the acute transfer of the drug from the stent, which is the most significant aspect of any drug‐coated device. It is noted that the minimal therapeutic levels of tissue paclitaxel have been reported in the range between 0.01 and 1 ng/mg.^42^, ^43^ Although this range should be maintained for up to 28 days, the most influential aspect of pharmacokinetics is the acute transfer of paclitaxel from the device to the tissue since the majority of drug loss occurs in the first hours and days.^44^ Figure 6 shows the presence of the paclitaxel drug on the luminal surface of the artery.

The results of our studies also demonstrated a significant loss of drug from the stent acutely. Based on the manufacturer's website, the deployed Zilver stent (6 mm × 40 mm) utilized in this study has a nominal paclitaxel amount of 383,000 ng. At 30 min post‐deployment, only 3522 ng of paclitaxel remained on the stent platform, translating to a 99% drug loss at 30 min. By 24 h, approximately 358 ng of the drug remained on the stent, translating to a 99.9% loss at 24 h. These significant losses can be observed within the SEM images, as at baseline (prior‐to deployment), large drug depositions can be observed within the stent struts, but not at later time points of 30 min and 24 h (Figure 5). In comparison to published data, Dake et al. showed that 5% of drug remained on the stent at 24 h in a pig peripheral model, translating to a 95% drug loss.^28^ They also demonstrated peak tissue drug concentration was measured to be 70.7 ± 21.2 ng/mg, similar to our results at 30 min (119 ± 26.1 ng/mg). It is noted that the study by Dake et al. was published in 2011, a year before the FDA‐approval of the Zilver stent. We are not aware of any other publications on the pharmacokinetics of the Zilver stent.

Some limitations of our system include that the working fluid is culture medium rather than whole blood, and that the current studies only used healthy arteries (not diseased). Despite these limitations, evaluation of drug delivery, elution, and retention can be accomplished. Utilizing our system, off‐the‐shelf vascular devices can be deployed in an ex vivo native artery, conditioned at physiological mechanical conditions, and evaluated for drug pharmacokinetics.

## 5 CONCLUSION

This system represents the first ex vivo pulsatile system to determine pharmacokinetics in native blood vessels undergoing peripheral vascular motion. The system's ability to mimic the bending, twisting, and shortening–elongating of an artery allows the system to reduce the time and expense associated with in vivo testing of vascular devices, particularly in measuring and quantifying vessel drug retention within the periphery. Future studies will include longer time points, more severe vascular motion, and monitoring smooth muscle cell proliferation, endothelialization, and other biomarkers to understand the acute response of explanted arteries to arterial drug delivery systems. More advances in this type of technology will undoubtedly continue with the hope of reducing the preclinical trial expense and the time‐to‐market of many vascular devices.

## AUTHOR CONTRIBUTIONS

**Danyi Chen:** Conceptualization (equal); data curation (equal); formal analysis (equal); methodology (equal); software (equal); writing – original draft (equal); writing – review and editing (equal). **Colin Krinsky:** Data curation (equal); formal analysis (equal); methodology (equal); software (equal); writing – review and editing (equal). **Mollie Phillips:** Data curation (equal); methodology (equal); writing – review and editing (equal). **Catherine Allred:** Data curation; methodology; writing – review and editing. **Ava Khan:** Data curation (equal); writing – review and editing (equal). **Linda Liu:** Data curation (equal); writing – review and editing (equal). **Uwe Christians:** Data curation (equal); writing – review and editing (equal). **Saami Yazdani:** Conceptualization (lead); data curation (lead); formal analysis (lead); funding acquisition (lead); investigation (lead); methodology (lead); project administration (lead); resources (lead); supervision (lead); writing – original draft (lead); writing – review and editing (lead).

## FUNDING INFORMATION

This work was supported in part by the National Institute of Health [#1R01EB028798].

## CONFLICT OF INTEREST STATEMENT

Saami K. Yazdani serves on the Scientific Advisory Board of Advanced Catheter and has received grant support from Advanced Catheter Therapies, Alucent Biomedical, Toray Industries, Biosensors International, Advanced NanoTherapies, OrbusNeich Medical and BD. All other authors have no conflicts of interest to declare.

### PEER REVIEW

The peer review history for this article is available at https://www.webofscience.com/api/gateway/wos/peer-review/10.1002/btm2.10618.

## Supporting information


Video S1.

## Data Availability

The data supporting the findings of this study are available from the corresponding authors upon reasonable request.
